# The new generation PFAS C6O4 does not produce adverse effects on thyroid cells in vitro

**DOI:** 10.1007/s40618-020-01466-4

**Published:** 2020-12-14

**Authors:** F. Coperchini, L. Croce, P. Pignatti, G. Ricci, D. Gangemi, F. Magri, M. Imbriani, M. Rotondi, L. Chiovato

**Affiliations:** 1 Unit of Internal Medicine and Endocrinology, Laboratory for Endocrine Disruptors, Istituti Clinici Scientifici Maugeri IRCCS, Via S. Maugeri 4, 27100 Pavia, Italy; 2 PHD Course in Experimental Medicine, University of Pavia, 27100 Pavia, Italy; 3 Allergy and Immunology Unit, Istituti Clinici Scientifici Maugeri IRCCS, 27100 Pavia, Italy; 4 Department of Public Health, Experimental and Forensic Medicine, University of Pavia, 27100 Pavia, Italy; 5 Department of Internal Medicine and Therapeutics, University of Pavia, 27100 Pavia, Italy

**Keywords:** C6O4, PFOA, PFOS, Perfluoroalkyl substances, Thyroid, FRTL5, Endocrine disruptors

## Abstract

**Purpose:**

Per- and poly-fluoroalkyl-substances (PFASs) are synthetic compounds that raised concern due to their potential adverse effects on human health. Long-chain PFAS were banned by government rules in many states, and thus, new emerging PFAS were recently introduced as substitutes. Among these, Perfluoro{acetic acid, 2-[(5-methoxy-1,3-dioxolan-4-yl)oxy]}, ammonium salt (C6O4) was recently introduced to produce a range of food contact articles and literature data about this compound are scanty. The aim of this study was to evaluate the in vitro effects of exposure to C6O4, compared with PFOA and PFOS on thyroid cells.

**Methods:**

FRTL5 rat-thyroid cell lines and normal human thyroid cells (NHT) were incubated with increasing concentrations of C6O4 for 24, 48, 72, and 144 h to assess cell viability by WST-1. Cell viability was confirmed by AnnexinV/PI staining. Long-chain PFAS (PFOA and PFOS) were used at same concentrations as positive controls. The proliferation of cells exposed to C6O4, PFOA, and PFOS was measured by staining with crystal violet and evaluation of optical density after incubation with SDS. Changes in ROS production by FRTL5 and NHT after exposure to C6O4 at short (10, 20, and 30 min) and long-time points (24 h) were evaluated by cytofluorimetry.

**Results:**

C6O4 exposure did not modify FRTL5 and NHT cell viability at any concentration and/or time points with no induction of necrosis/apoptosis. At difference, PFOS exposure reduced cell viability of FRTL5 while and NHT, while PFOA only in FRTL5. FRTL5 and NHT cell proliferation was reduced by incubation with by PFOA and PFOS, but not with C6O4. ROS production by NHT and FRTL5 cells was not modified after C6O4 exposure, at any time/concentration tested.

**Conclusions:**

The present in vitro study constitutes the first evaluation of the potential adverse effects of the new emerging PFAS C6O4 in cultured rat and human thyroid cells, suggesting its safety for thyroid cells in vitro.

## Introduction

Per- and poly-fluoroalkyl substances (PFASs) are a class of man-made chemicals, globally used as surfactants in industrial productions due to their surface active properties as well as to their thermal and chemical stability [1]. Among PFAS, two “Long chain PFAS” (so-called owing to the presence of 8 or more carbon atoms), perfluorooctanoic acid (PFOA) and perfluorooctanesulfonic acid (PFOS), were object of growing concerns due to their elevated persistency and bioaccumulation in the environment [2]. Indeed, these compounds are constantly discharged into the environment from manufacturing processes and daily usage, being persistent and with long time of degradation [1].

Further concern derives from the fact that PFOA and PFOS were reported to act as endocrine thyroid disruptors and, in some cases, as cancer promoting agents [3]; they also induce oxidative stress, both in humans and in animal models [3–6]. Due to their potential adverse effects for human health, long-chain PFAS production was restricted or even banned in some countries [7]. With the aim to reduce the ecological and health impact of long-chain PFAS, industries availed of both short-chain alternatives as well as of newly synthesized PFAS [8]. Whether these alternative compounds should be really considered safe for human health and for the environment remains to be fully elucidated [9]. Several studies highlighted toxic effects of some short-chain PFAS compounds in different types of cells [10, 11]). A recent study also demonstrated that a new generation PFAS, i.e., hexafluoropropylene-oxide-dimer-acid (commonly known as GenX), exerts in vitro cytotoxic and genotoxic effects on thyroid cells [12]. On the other hand, other short-chain PFAS, such as perfluorobutanesulfonic acid (PFBS), perfluorobutanoic acid (PFBA), pentafluoropropionic anhydride (PFPA), and perfluoropentanoic acid (PFPeA), did not affect thyroid cell viability [13]. Another PFAS, perfluoro acetic acid, 2-[(5-methoxy-1,3-dioxolan-4-yl)oxy], ammonium salt (commonly known as C6O4) was recently introduced in the polymerization process of fluoropolymers to produce a range of food contact articles such as fittings and valves [14]. C6O4 has a high degree of solubility (> 667 g/L at 21 °C) and is thermally unstable. It is manufactured and/or imported in the European Economic Area in 1–10 tons per year [15]. On 16 April 2019, ARPAV (the Regional Agency for the Prevention and Protection of the Environment in Veneto) detected C6O4 in the River Po near certain municipalities in the Veneto region, bordering on the Emilia-Romagna region (Corbola), and in one municipality on the border between Lombardy and Emila-Romagna regions (Castelmassa), with peak concentrations of around 100 ng/l [16].

Previous in vitro studies investigated the toxic effects of several PFAS in different types of cultured cells including thyroid cells [3, 17–21]. However, no information is still available regarding the in vitro effects of C6O4 on thyroid cells. Aim of the present study was to evaluate the in vitro effects of C6O4 compared with PFOA and PFOS on a strain of differentiated rat-thyroid cells (FRTL5 cells) and on primary cultures of normal human thyroid cells (NHT) in terms of cell viability, proliferation rate, and reactive oxygen species (ROS) production, after both short and long time of exposure.

## Materials and methods

### Cultures of FRTL5 cells

The differentiated strain of rat-thyroid cells (Fisher-rat-thyroid-line-5; FRTL5; American Type Culture Collection, Manassas, VA; ATCC CRL 8305, F1 subclone) served as an in vitro experimental substrate. Cells were grown for 1 week in 6H medium (Coon’s Modified Ham’s F-12 Medium (Sigma Chemical Co.) supplemented with 5% adult calf serum (BioWhittaker, Inc., Walkersville, MD) and a six-hormone mixture containing insulin, somatostatin, hydrocortisone, transferrin, glycyl-histidyl-lysine, and TSH (Sigma Chemical Co.).

### Primary cultures of human thyroid cells

Surgical specimens of normal human thyroid were obtained from the contralateral disease-free lobe of patients who underwent thyroidectomy for a solitary benign nodule (*n* = 3). Surgical specimens were minced and then incubated with collagenase type II (Sigma, Saint Louis, MO, USA) 5 mg/ml, in 5 ml of Coon’s F12 medium, for 4 h at 37 °C. Then, 10 ml of Coon’s F12 medium were added, following which, cells were filtered, spun at 1000 × g for 10 min, washed with Coon’s F12 medium, spun again, and finally re-suspended in complete medium containing 5% newborn calf serum and a mixture of six hormones including insulin (5 μg/ml), hydrocortisone (50 μg/ml), transferrin (5 μg/ml), somatostatin (10 ng/ml), gly-his-lysine (10 ng/ml), and bovine TSH (1 mU/ml).

### Cell viability and WST-1 assay

FRTL5 and NHT cells were grown until an 80% confluence was reached. Thereafter, cells were detached and seeded in 96-well flat plates at a density of 2 × 10^4^ cell/well. 6H medium was supplemented with C6O4, PFOA, or PFOS at the following concentrations: 0, 0.01, 0.1, 1, 10, and 100 ng/ml. Because no information is currently available regarding the serum concentrations of C6O4 in humans, experiments were performed using a range of compound concentrations which corresponded to that observed for other long- and short-chain PFAS in the general population and in exposed workers [13]. The incubation times of FRTL5 and NHT with C6O4 were 24, 48, 72, and 144 h. Control FRTL5 and NHT cells were cultured in plain 6H medium. At the end of each time point, 20 μl of WST-1 were added to each well; plates were then incubated for 30 min at 37° in a 5% CO_2_ atmosphere. WST-1 is a colorimetric reagent, which, after cleavage of a tetrazolium salt by mitochondrial dehydrogenases, results in the production of formazan if the cell is viable. A reduction in the overall activity of mitochondrial dehydrogenases also decreases the amount of produced formazan. Using a multimode plate reader at the absorbance of 450 nm (Victor NIVO Multimode Microplate Reader, PerkinElmer), we quantified the amount of produced formazan.

### Annexin V-FITC/PI assay to detect programmed cell death (apoptosis), late apoptotic, or necrotic cells

The use of AnnexinV/PI staining allows discriminating live cells from apoptotic (green fluorescence, Annexin V positive cells), necrotic (red fluorescence, PI positive cells), or late apoptotic (green and red fluorescence, AnnexinV and PI positive cells) cells. During apoptosis, the normal asymmetry of the phospholipidic membrane is destroyed and phosphatidylserine (a component of the internal surface of the cell membrane) is exposed on the outside surface of the plasma membrane [22]. Since Annexin V protein binds with high affinity to phosphatidylserine, fluorochrome-conjugated Annexin V (displaying a green fluorescence) is commonly used to detect apoptotic cells. The differentiation between apoptotic and late apoptotic or necrotic cells is performed by simultaneous staining with Propidium Iodide (PI), a fluorescent DNA-intercalating agent. PI penetrates in cells when the integrity of their plasma and nuclear membranes is impaired [23]. This is typical of late apoptotic and necrotic cells. PI intercalates into nucleic acids and displays a red fluorescence [24].

The cell plasma membrane exposure of phosphatidylserine was assessed using Annexin V-FITC Apoptosis Detection Kit (Life-Technologies Apo-Detect Kit). Briefly, FRTL5 and NHT cells were harvested at a density of 10^4^ cells per well on a coverslip placed in a 24-well plate. After adhesion, cells were exposed to the highest concentration of C6O4, PFOA, or PFOS. After 144 h of exposure, cells were washed with phosphate buffer saline (PBS) and cell culture supernatants were stored for subsequent cyto-spin. Coverslips were incubated with a mix of Annexin V-FITC, PI, and Hoechst 33,258 (Hoecst is a blue fluorescent dye which stains DNA in nuclei) (Thermofisher) in the dark for 10 min at room temperature. The same procedure was performed on cells recovered in the supernatants after cyto-spin. Cells were fixed with PFA 4% for 10 min. After washing with PBS, coverslips were mounted with Dako (cells recovered by cyto-spin were placed onto a slide and covered with a coverslip also mounted with Dako) and fluorescence images were obtained with Olympus IX83 fluorescent inverted microscope.

### Cell proliferation assay

FRTL5 and NHT cells were seeded in a 12-well plate at a density of 500 cells per well and incubated in the presence or absence of increasing concentrations of C6O4, PFOA, or PFOS (0, 0.01, 0.1, 1, 10, and 100 ng/ml) for 6 days. Cells were fixed with methanol for 20 min and stained with 0.5% crystal violet dye (which binds to proteins and DNA of viable cells conferring them a violet staining) for 5 min [25]. Cell proliferation was assessed by observation with an Olympus BX51 inverted microscope (Olympus, Deutschland GmbH, Hamburg, Germany). For quantification of proliferation, cells were washed three times with deionized water to remove excess of stain. Subsequently, cells were incubated for 2 h with 1% Sodium dodecyl sulfate (SDS). The incubation with SDS led the crystal violet dye to be released from cells into the supernatant. The measured (at 570 nm using a Victor NIVO Multimode Microplate Reader) crystal violet in the supernatant is proportional to the number of cells per well.

### Reactive oxygen species (ROS) production

Generation of cellular Reactive Oxygen Species (ROS) is induced by both endogenous and exogenous stimuli. ROS are formed as a natural bio-products of the normal oxygen metabolism, and have important roles in homeostasis and cell signaling. In most cell types, they are also involved in the damage of cell structures and mitochondrial function [26].

The cell-permeant 2′, 7′-dichlorodihydrofluorescein diacetate (H2DCFDA) was used to assess reactive oxygen species (ROS) production by FRTL5 and NHT treated with C6O4. H2DCFDA is a fluorogenic dye that measures, within the cell, hydroxyl, peroxyl, and other ROS activity. This dye, after diffusion into the cell, is deacetylated by cellular esterases to a non-fluorescent compound, which is later oxidized by ROS into a high fluorescent compound 2′, 7′-dichlorofluorescein (DCF) [27].

After seeding in a 24-well plate at a cell concentration of 5 × 10^4^ per well, FRTL5 and NHT cells were treated with C6O4 at increasing concentrations for short time points (10, 20, and 30 min) and a long time point (24 h). The choice of these time points stems from the intent of detecting both early and late ROS production. H2DCFDA was added to cultures for ten minutes after each time point of C6O4 exposure. Cells were then detached with a trypsin 0.05%, 0.002% Ethylene-diamine-tetra-acetic acid (EDTA) mixture, centrifuged and re-suspended in a solution of phosphate buffer saline/bovine serum albumin (PBS/BSA) for being analyzed by a flow cytometer (laser excitation 492 nM), and analyzed with Cell Quest software (FACScan, Becton Dickinson). FRTL5 and NHT cells treated with Ammonium persulphate 10 mMol were used as a positive control for ROS induction. The assay was performed in concomitance with PI staining to assess if ROS induction was associated with a cytotoxic effect, the death rate being expressed as a percentage of Mean Fluorescence Intensity of untreated cells.

### Statistical analysis

Statistical analysis was performed using the SPSS software (SPSS, Inc., Evanston, IL). Mean group values were compared using one-way ANOVA for normally distributed variables. Post hoc analysis was performed by Bonferroni’s correction for multiple comparisons. Values are reported as mean ± SD unless otherwise noted. A *p* value < 0.05 was considered statistically significant.

## Results

### Effect of C6O4 on FRTL5 and NHT cell viability

**Fig. 1:**
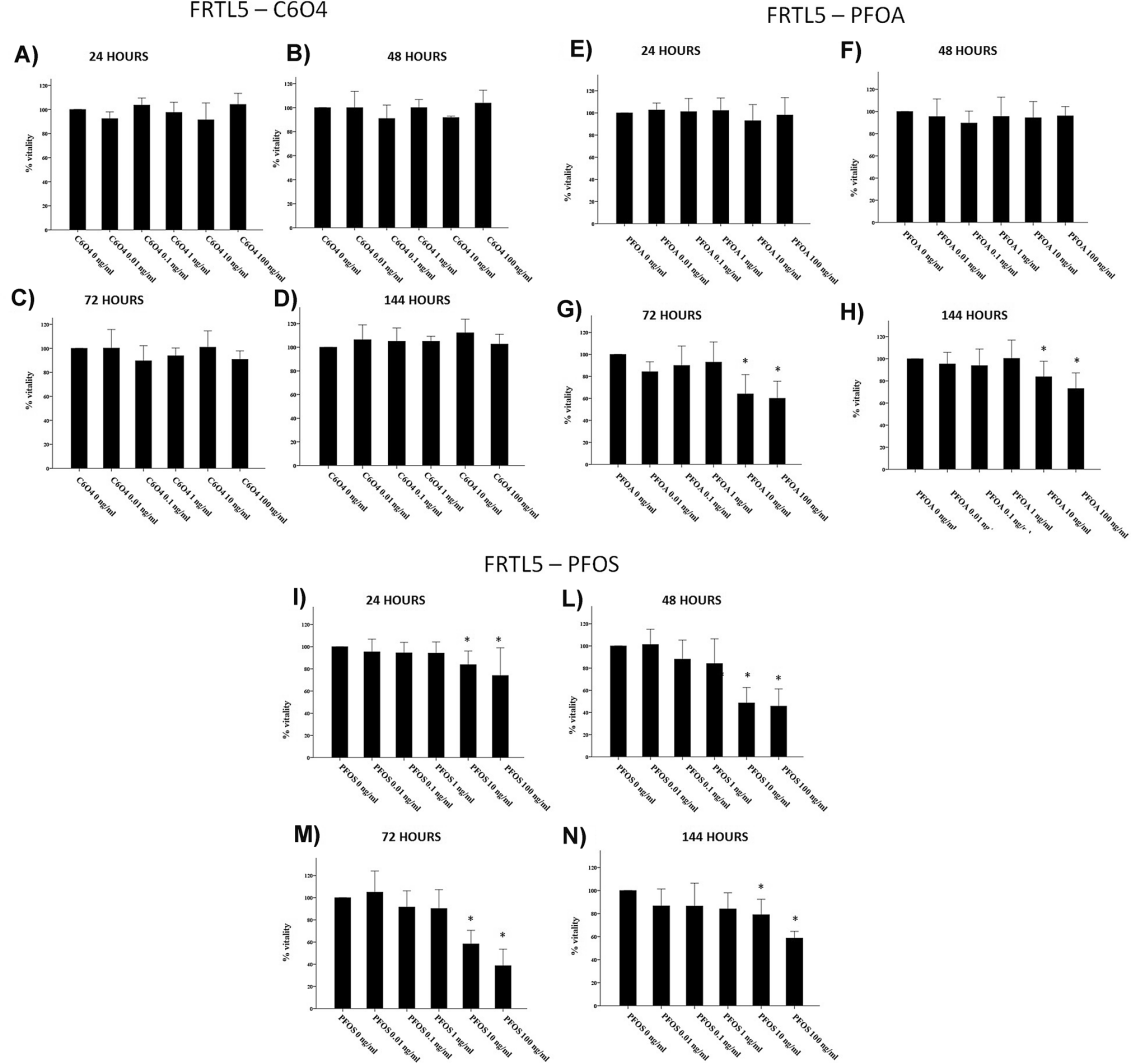
Effect of C6O4, PFOA, and PFOS exposure on FRTL5 viability. Incubation with C6O4 did not reduce cell viability at any concentration. **a** 24-h incubation (ANOVA *F* = 1.706; *p* = 0.184). **b** 48-h incubation (ANOVA *F* = 1.374, *p* = 0.280), **c** 72-h incubation (ANOVA *F* = 0.933, *p* = 0.483). **d** 144-h incubation (ANOVA: *F* = 1.615, *p* = 0.177) Exposure to PFOA reduced FRTL5 cells viability. **e** 24-h incubation (ANOVA: *F* = 1.439, *p* = 0.220). **f** 48-h incubation (ANOVA *F* = 1.021, *p* = 0.411). **g** 72-h incubation (ANOVA: *F* = 9.316, *p* < 0.001). **h** 144 h (ANOVA: *F* = 9.892, *p* < 0.0001). Exposure to PFOS reduced FRTL5 cells viability. **i** 24-h incubation (ANOVA: *F* = 7.252, *p* < 0.001). **l** 48-h incubation (ANOVA *F* = 37.392, *p* < 0.001). **m** 72-h incubation (ANOVA: *F* = 47.381, *p* < 0.001). **n** 144 h (ANOVA: *F* = 8.718, *p* < 0.0001). Results are expressed as percentage (%) of viable cells calculated on the OD of untreated samples estimated as 100%

**Fig. 2:**
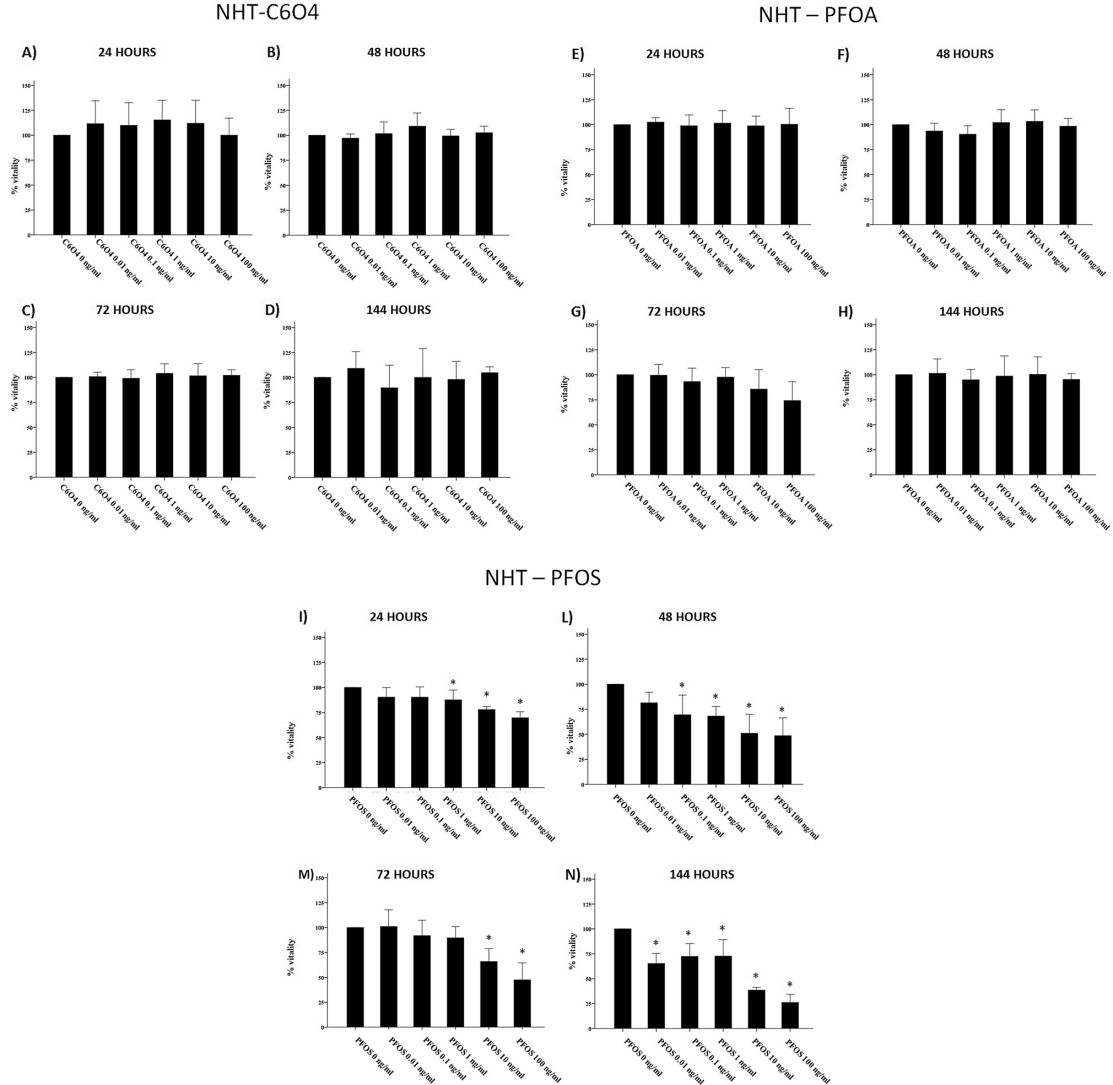
Effect of C6O4, PFOA, and PFOS exposure on NHT viability. Incubation with C6O4 did not reduce cell viability at any concentration. **a** 24-h incubation (ANOVA *F* = 0.917, *p* = 0.479). **b** 48-h incubation (ANOVA *F* = 1.978, *p* = 0.102), **c** 72-h incubation (ANOVA *F* = 0.393, *p* = 0.851), and **d** 144-h incubation (ANOVA: *F* = 1.050, *p* = 0.401). Incubation with PFOA did not reduce NHT cell viability. **e** 24-h incubation (ANOVA: *F* = 0.148, *p* = 0.979). **f** 48-h incubation (ANOVA: *F* = 2.227, *p* = 0.072). **g** 72-h incubation (ANOVA *F* = 3.641, *p* = 0.07). **h** 144-h incubation (ANOVA: *F* = 0.306, *p* = 0.906). Incubation with PFOS reduced NHT cell viability. **e** 24-h incubation (ANOVA: *F* = 15.338, *p* < 0.05). **f** 48-h incubation (ANOVA: *F* = 13.609, *p* < 0.001). **g** 72-h incubation (ANOVA: *F* = 18.239, *p* < 0.001). **h** 144-h incubation (ANOVA: *F* = 52.615, *p* < 0.001)

Exposure of FRTL5 to increasing concentrations of C6O4 did not affect thyroid cell viability at any concentration tested after 24, 48, 72, and 144 h (ANOVAs: 24 h *F* = 1.706; *p* = 0.184 48 h *F* = 1.374, *p* = 0.280; 72 h *F* = 0.933, *p* = 0.483; 144 h *F* = 1.615, *p* = 0.177) (Fig. 1a–d). The WST-1 assay indicated that not only the viability of FRTL5 was not affected by C6O4, but also that no damage on the mitochondrial respiration occurred following exposure to the compound. On the contrary, exposure of FRTL5 to increasing concentrations of PFOA or PFOS affected cell viability. In particular, a significant reduction of cell viability was registered after 72 and 144 h of exposure to PFOA (ANOVAs: 72 h *F* = 9.316, *p* < 0.001; 144 h *F* = 9.892, *p* < 0.0001) (Fig. 1e–h) and after all time of exposure to PFOS (ANOVAs: 24 h *F* = 7.252, *p* < 0.001; 48 h *F* = 37.392, *p* < 0.0001; 72 h *F* = 47.381, *p* < 0.0001; 144 h *F* = 8.718, *p* < 0.0001) (Fig. 1i–n). As for NHT cells, C6O4 exposure, similarly to FRTL5, did not affect cell viability at any time point and concentration tested (ANOVAs: 24 h *F* = 0.917, *p* = 0.479; 48 h *F* = 1.978, *p* = 0.102; 72 h *F* = 0.393, *p* = 0.851,; 144 h *F* = 1.050, *p* = 0.401) (Fig. 2a–d). Similarly, PFOA exposure did not modify NHT cell viability at all time points and concentrations (ANOVA *F* = 3.641, *p* = 0.07) (Fig. 2d–g) At difference, PFOS exposure induced a reduction of NHT cell viability, starting from different concentrations at all time points (ANOVAs 24 h *F* = 15.338, *p* < 0.05; 48 h *F* = 13.609 *p* < 0.05; 72 h *F* = 18.239 *p* < 0.05; 144 h *F* = 52.615 *p* < 0.05) (Fig. 2h–m).

**Fig. 3:**
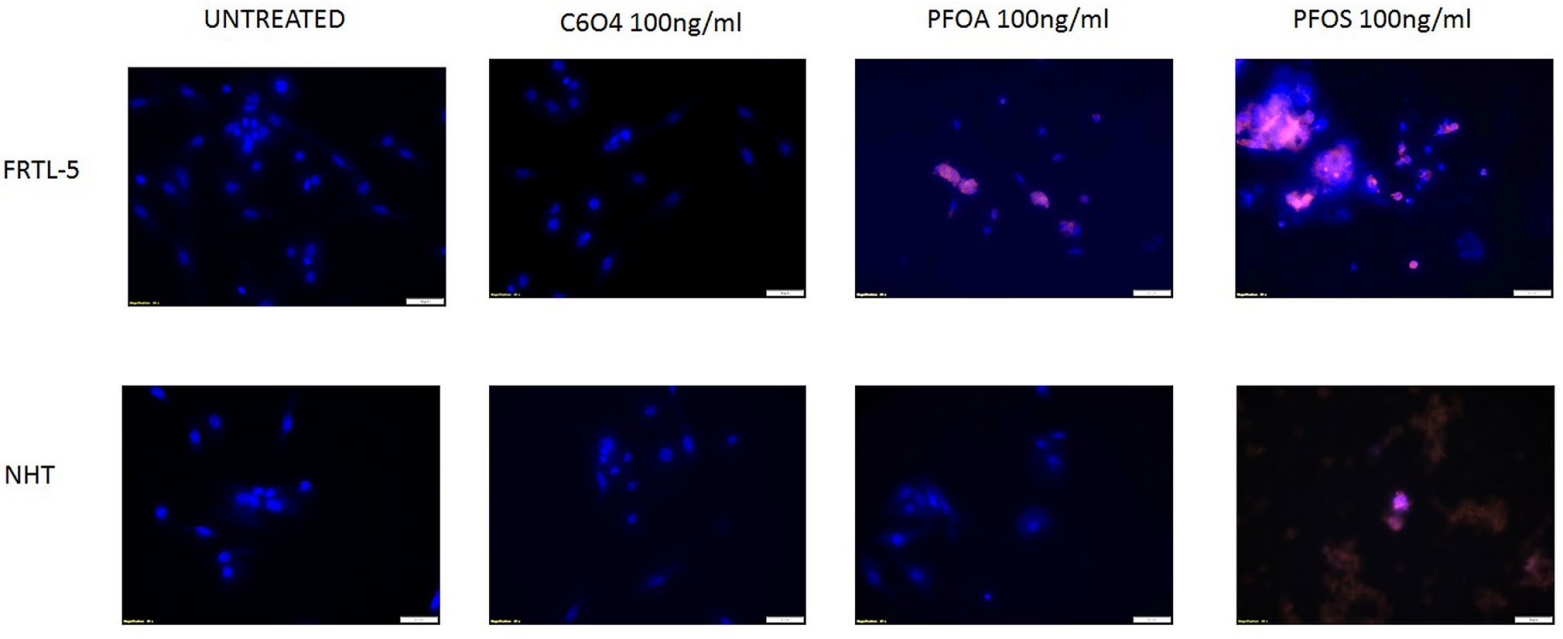
Evaluation of induced apoptosis in FRTL5 and NHT cells (Annexin V-FITC/PI stain under fluorescent microscope (×20 magnification). Cells were treated up-top 144 h with medium alone (C6O4, PFOA, PFOS = 0 ng/ml) or with C6O4, PFOA, or PFOS (100 ng/ml). Representative images of merged Annexin V/PI/Hoecst in three experiments are shown. Both control, untreated FRTL5 and NHT cells and cells treated with C6O4 at 100 ng/ml were positive only for Hoecst staining of the nuclei. FRTL5 cells treated with PFOA and PFOS were positive to the fluorescent bright orange-green stain of Annexin V-FITC/PI. NHT cells were positive for treatment with PFOA but not for treatment with PFOS

To further confirm data on cell viability, the AnnexinV/PI assay was performed. FRTL5 and NHT incubated for 144 h with 100 ng/ml C6O4 did not show changes in cell apoptosis and/or necrosis. At difference, the same concentration of PFOA and PFOS induced cell death in FRTL5 while PFOS but not PFOA induce cell death in NHT. Figure 3 shows representative images of FRTL5 and NHT cells exposed for 144 h to the highest concentration of C6O4, PFOA, and PFOS (100 ng/ml).

### Effects of C6O4 on FRTL5 cell proliferation

**Fig. 4:**
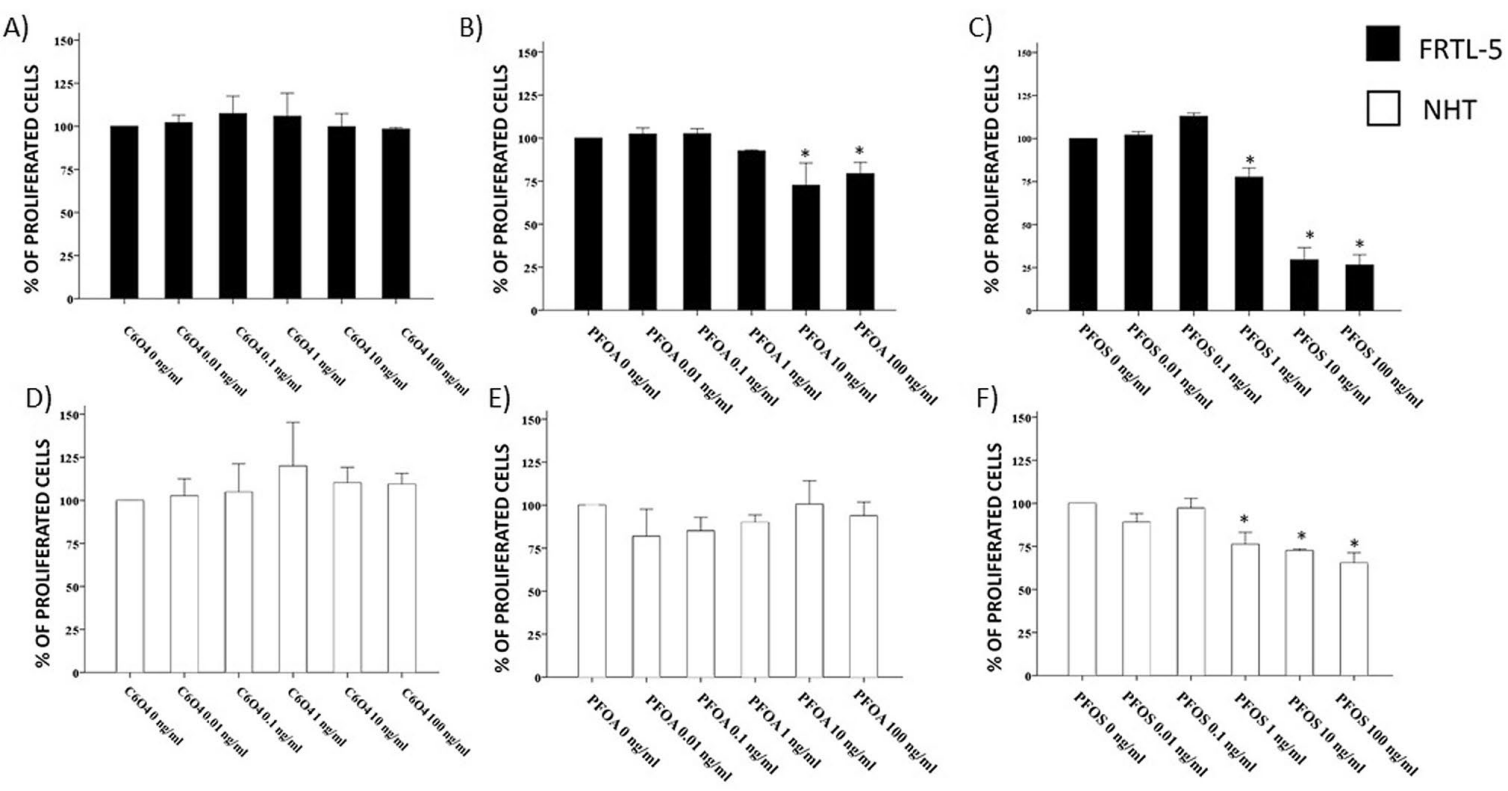
Assay of cell proliferation in FRTL5 and NHT following exposure to C6O4, PFOA, and PFOS. **a** The ability of FRTL5 cells to proliferate was not reduced after 6 days at any concentration tested (ANOVA *F* = 0.648 *p* = 0.668). **b**, **c** Exposure of FRTL5 to PFOA and PFOS showed a reduction of cells proliferation (ANOVA PFOA: *F* 12.881 *p* < 0.05; ANOVA PFOS: *F* = 218.105 *p* < 0.001). **d** The ability of NHT to proliferate was not reduced by C6O4 (ANOVA *F* = 0.809 *p* = 0.565). **e**, **f** Exposure of NHT cells to PFOA did not show changes in cell proliferation (ANOVA *F* = 1.860 *p* = 0.176) at difference with PFOS (ANOVA *F* = 25.566 *p* < 0.001)

Exposure to increasing concentrations of C6O4 for 6 days did not affect thyroid cell proliferation at any concentration tested in both FRTL5 (ANOVA *F* = 0.648 *p* = 0.668) (Fig. 4a) and in NHT cells (ANOVA *F* = 0.809 *p* = 0.565) (Fig. 4d). Exposure of FRTL5 to PFOA and PFOS showed a reduction of cell proliferation (ANOVA PFOA: *F* 12.881 *p* < 0.05; ANOVA PFOS: *F* = 218.105 *p* < 0.001) (Fig. 4b, c). Exposure of NHT cells to PFOA did not show changes in cell proliferation (ANOVA *F* = 1.860 *p* = 0.176) at difference with PFOS that reduced cell proliferation (ANOVA *F* = 25.566 *p* < 0.001) (Fig. 4e, f).

### Short and long time exposure to C6O4 and induction of ROS in FRTL5 cells and NHT

**Fig. 5:**
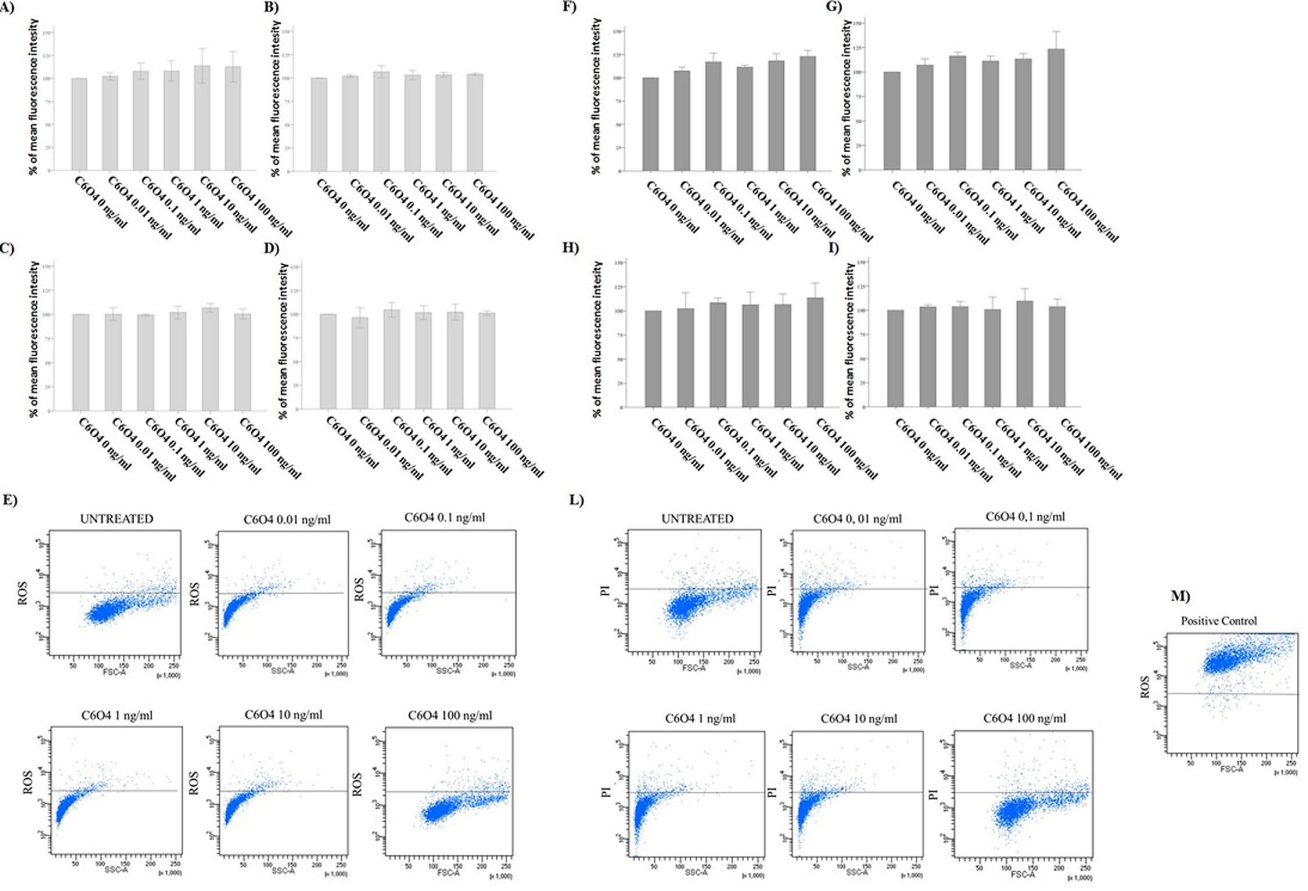
Assay of ROS production in FRTL5 cells (determined using the H2DCFDA assay) paralleled by PI staining using FACS. Results are expressed as percentage (%) of the mean fluorescence intensity of untreated controls. ROS production did not increase after exposure to C6O4 at any concentration. Histogram **a** after 10-min exposure (ANOVA *F* = 0.435, *p* = 0.810). **b** after 20-min exposure (ANOVA *F* = 1.168, *p* = 0.379). Histogram **c** after 30-min exposure (ANOVA *F* = 0.432, *p* = 0.818). Histogram **d** after 24-h exposure (ANOVA *F* = 0.665, *p* = 0.665). **e** representative images of ROS production determined with H2DCFDA at flow cytometry analysis in FRTL5 cells. Images shows untreated cells and cells exposed to C6O4 at increasing concentrations. PI staining did not increase after exposure to C6O4 at any concentration Histogram **f** after 10-min exposure (ANOVA *F* = 0.033, *p* = 0.060). Histogram **g** after 20-min exposure (ANOVA *F* = 2.690, *p* = 0.074). Histogram* h* after 30-min exposure (ANOVA *F* = 0.478, *p* = 0.786). Histogram* i* after 24-h exposure (ANOVA *F* = 0.325, *p* = 0.881). **l** representative images PI + cells determined by flow cytometry analysis in FRTL5 cells. Images show untreated cells and cells exposed to C6O4 at increasing concentrations. **m** Representative images of positive control. Ammonium persulphate was used as positive control for ROS induction

**Fig. 6:**
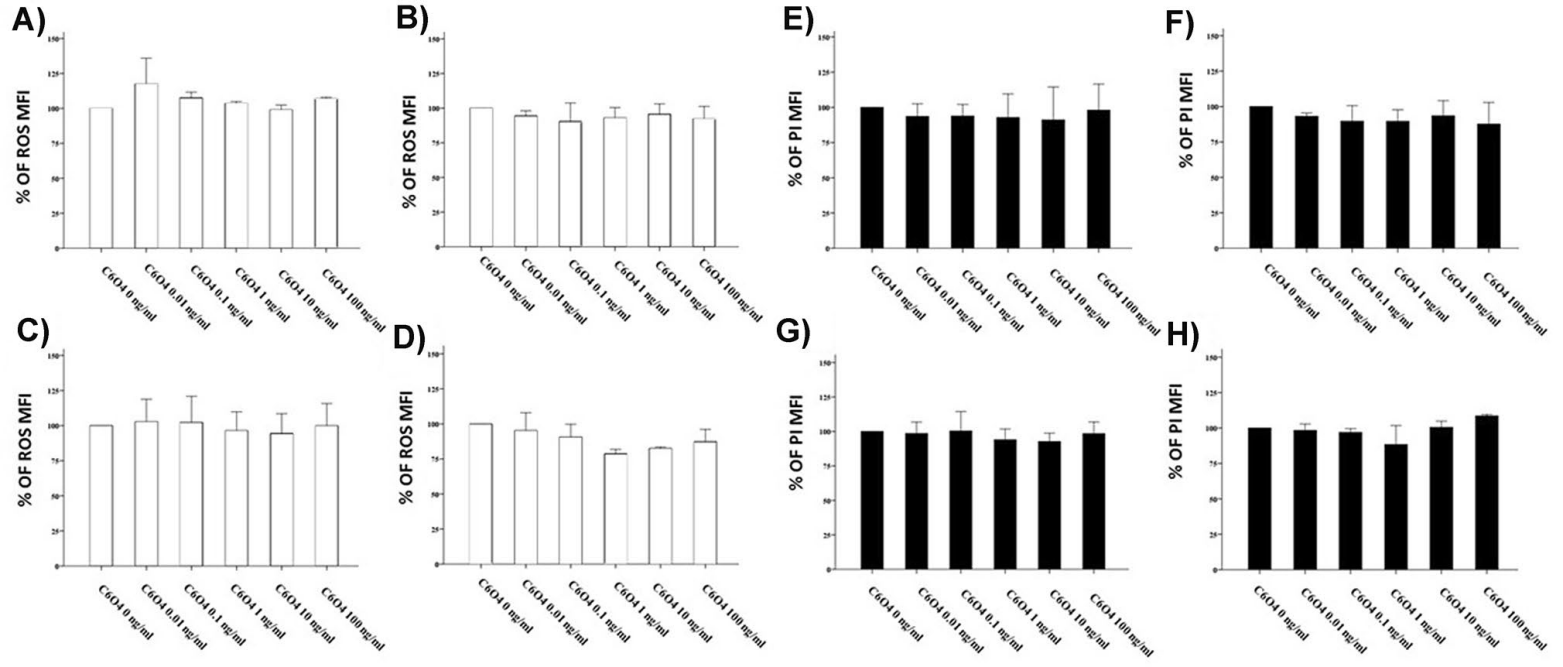
Assay of ROS production in NHT cells (determined using the H2DCFDA assay) paralleled by PI staining using FACS. Results are expressed as percentage (%) of the mean fluorescence intensity of untreated controls. ROS production did not increase after exposure to C6O4 at any concentration. Histogram **a** after 10-min exposure (ANOVA *F* 1.47 *p* = 0.324). **b** after 20-min exposure (ANOVA *F* 0.344 *p* = 0.869). Histogram **c** after 30-min exposure (ANOVA *F* 0.107 *p* = 0.987). Histogram **d** after 24-h exposure (ANOVA *F* 2.248 *p* = 0.176). PI staining did not increase after exposure to C6O4 at any concentration Histogram Panel E: after 10-min exposure (ANOVA *F* 0.104 *p* = 0.988). Histogram **f** after 20-min exposure (ANOVA *F* 0.435 *p* = 0.811). Histogram **g** after 30-min exposure (ANOVA *F* 0.282 *p* = 0.907). Histogram **h** after 24-h exposure (ANOVA *F* 2.256 *p* = 0.175)

C6O4 exposure for short (10, 20, or 30 min) and long time (24 h) did not induce ROS production in FRTL5cells at any concentration. This was consistently observed at any time point (10 min: ANOVA *F* = 0.435, *p* = 0.810; 20 min ANOVA *F* = 1.168, *p* = 0.379; 30 min: ANOVA *F* = 0.432, *p* = 0.818, 24 h: ANOVA *F* = 0.665, *p* = 0.665) (Fig. 5a–d). To rule out that the ROS production could be due to the induction of minimal cell death (in particular for short time points), PI positivity was assessed. This assay further confirmed the absence of any cytotoxic effect of C6O4 at any concentration and time point (10 min: ANOVA *F* = 0.033, *p* = 0.060; 20 min: ANOVA *F* = 2.690, *p* = 0.074; 30 min: ANOVA *F* = 0.478, *p* = 0.786; 24 h ANOVA *F* = 0.325, *p* = 0.881) (Fig. 5e–h). The exposure to C6O4 did not modify ROS production also in NHT cells at any time points (10 min: ANOVA, *F* 1.47 *p* = 0.324; 20 min ANOVA *F* 0.344 *p* = 0.869; 30 min: ANOVA *F* 0.107 *p* = 0.987 24 h: ANOVA *F* = 2,248 , *p* = 0,176) (Fig. 6a–d). The assessment of PI positivity further confirmed the absence of cellular death (10 min: ANOVA *F* 0.104 *p* = 0.988; 20 min: ANOVA *F* 0.435 *p* = 0.811; 30 min: ANOVA *F* = 0.282 *p* = 0.907; 24 h ANOVA) (Fig. 6e–h).

## Discussion

The present study, for the first time, evaluates the potential in vitro adverse effects of a new PFAS, C6O4, in comparison with long-chain PFAS, PFOA, and PFOS, in cultured rat and human thyroid cells. Several end-points were taken into account, including cell viability, proliferation, induction of apoptosis, and ROS production. The results obtained showed that C6O4 has no detectable toxic effect on cultured thyroid cells. In particular, treatment with C6O4 did not affect FRTL5 cells viability as assessed either by mitochondrial respiration products (WST-1 assay) or by evaluation of apoptosis/necrosis (AnnexinV/PI staining) up to 144 h of exposure. At difference, long-chain PFAS, PFOA and mainly PFOS, both reduced cell viability of rat-thyroid cells. Slightly different results were found in NHT, in which C6O4 and PFOA did not modify cell viability at any time and concentration tested, at difference with PFOS, which exerted a significant cytotoxic effect at different concentrations and at any time point. The lack of mitochondrial damage in the WST-1 assay further supports the absence of a cytotoxic effect of C6O4 in both rat and human cultured thyroid cells. This is because WST-1 reacts with products of the mitochondrial respiration chain and serves as marker of their functionality [28]. The fact that mitochondrial activity would be preserved during C6O4 exposure is also supported by the observation that there was no ROS induction neither in FRTL5 nor in NHT. Mitochondria are believed to be the major intracellular source of ROS [29–32] and mitochondria-generated ROS are involved in physiologic signaling cascades, which regulates various cell and organ functions. However, in pathologic conditions, an excess production of mitochondrial ROS causes significant damage to cell structures leading to cell death [33–35]. Finally, C6O4 did not affect the proliferation rate of both FRTL5 and NHT cells. On the other hand, PFOA and PFOS, both reduced cells proliferation of FRTL5 while only PFOS but not PFOA also of NHT.

Although few previous data regarding the in vivo* and *in vitro effects of C6O4 exposure are available, it is worth attempting a comparison between previous and present findings. The ECHA registration dossier reports that C6O4 is free of in vivo acute toxic effects, as assessed by lack of mortality or other visible abnormalities in zebra fish after 96 h of exposure. Similarly, C6O4, at the concentration of 100 mg/L, did not affect the growth of aquatic algae and cyanobacteria (average growth rate and yield) throughout a 72-h test period. In addition, a scientific opinion published by EFSA reported that C6O4 does not induce gene mutations in mouse lymphoma cells and in bacteria, nor produces chromosome aberrations in rat bone marrow cells, as assessed by three in vitro and, more importantly, by one in vivo genotoxicity tests [14]. Similarly, the main decomposition product of C6O4 did not promote genetic alterations in bacteria and in Chinese hamster V79 cells [15]. Although potentially reassuring, these previous and present findings do not allow drawing firm general conclusions as to the safety of C6O4 exposure, but still they should be regarded as mandatory steps for further characterization of the safety profile of this new PFAS.

From a thyroid point of view, human studies reported that significant concentrations of PFOA and PFOS can be detected in thyroid gland surgical specimens [21]**.** Reassuringly, there is no evidence for an active thyroid concentration process both in vivo and in vitro [20]**.** In vitro experiments also showed that these compounds exert a thyroid cytotoxic effects only at very high concentrations [20]. The here reported results would confirm previous findings, that among long-chain PFAS, PFOS displays a more powerful toxic effect as compared to PFOA.

On the other hand, some short-chain PFAS do not exert in vitro toxic effects or alterations in the functional response to TSH of thyroid cells [13]. At variance with these reassuring results, another new generation PFAS, GenX was recently shown to exert both cytotoxic and genotoxic effects in cultured thyroid cells [12]. Taken together, these data indicate that PFAS, even if similar in chemical structure and properties, are profoundly different in terms of their toxicity profile, at least in thyroid cells.

The main limitation of the present study derives from the fact that there are no reported data on the serum or tissue levels of C6O4 in the general population or in exposed workers. Thus, the concentrations of C6O4 used in our in vitro experiments were chosen based on human data reported for other PFAS. Finally, although reassuring, the results of the present study require confirmation both in other types of animal and human cells and in in vivo models.

## References

[1] Buck RC, Franklin J, Berger U (2011). Perfluoroalkyl and polyfluoroalkyl substances in the environment: terminology, classification, and origins. Integr Environ Assess Manag.

[2] Wang Z, Cousins IT, Scheringer M, Buck RC, Hungerbühler K (2014). Global emission inventories for C4–C14 perfluoroalkyl carboxylic acid (PFCA) homologues from 1951 to 2030, Part I: production and emissions from quantifiable sources. Environ Int.

[3] Coperchini F, Awwad O, Rotondi M, Santini F, Imbriani M, Chiovato L (2017). Thyroid disruption by perfluorooctane sulfonate (PFOS) and perfluorooctanoate (PFOA). J Endocrinol Invest.

[4] Abdellatif A, Al-Tonsy AH, Awad ME, Roberfroid M, Khan MN (2003). Peroxisomal enzymes and 8-hydroxydeoxyguanosine in rat liver treated with perfluorooctanoic acid. Dis Markers.

[5] Panaretakis T, Shabalina IG, Grandér D, Shoshan MC, DePierre JW (2001). Reactive oxygen species and mitochondria mediate the induction of apoptosis in human hepatoma HepG2 cells by the rodent peroxisome proliferator and hepatocarcinogen, perfluorooctanoic acid. Toxicol Appl Pharmacol.

[6] Liu C, Yu K, Shi X (2007). Induction of oxidative stress and apoptosis by PFOS and PFOA in primary cultured hepatocytes of freshwater tilapia (Oreochromis niloticus). Aquat Toxicol.

[7] Secretariat of the Stockholm Convention: The new POPs under the Stockholm Convention (2011). https://chm.pops.int/Implementation/NewPOPs/ThenewPOPs/tabid/672/Default.aspx

[8] Xiao F (2017). Emerging poly- and perfluoroalkyl substances in the aquatic environment: a review of current literature. Water Res.

[9] Gomis MI, Vestergren R, Borg D, Cousins IT (2018). Comparing the toxic potency in vivo of long-chain perfluoroalkyl acids and fluorinated alternatives. Environ Int.

[10] Li J, He J, Niu Z, Zhang Y (2020). Legacy per- and polyfluoroalkyl substances (PFASs) and alternatives (short-chain analogues, F-53B, GenX and FC-98) in residential soils of China: present implications of replacing legacy PFASs. Environ Int.

[11] Bjork JA, Wallace KB (2009). Structure-activity relationships and human relevance for perfluoroalkyl acid-induced transcriptional activation of peroxisome proliferation in liver cell cultures. Toxicol Sci.

[12] Coperchini F, Croce L, Denegri M (2020). Adverse effects of in vitro GenX exposure on rat thyroid cell viability, DNA integrity and thyroid-related genes expression. Environ Pollut.

[13] Croce L, Coperchini F, Tonacchera M, Imbriani M, Rotondi M, Chiovato L (2019). Effect of long- and short-chain perfluorinated compounds on cultured thyroid cells viability and response to TSH. J Endocrinol Invest.

[14] EFSA (2014) Scientific Opinion on the safety assessment of the substance, Perfluoro{acetic acid, 2-[(5-methoxy-1,3-ioxolan-4-yl)oxy]}, ammonium salt, CAS No 1190931-27-1, for use in food contact materials. In. Vol 12, EFSA JOURNAL, p 3718. https://www.efsa.europa.eu/en/efsajournal/pub/3718

[15] ECHA (2009) Difluoro{[2,2,4,5-tetrafluoro-5-(trifluoromethoxy)-1,3-dioxolan-4-yl]oxy}acetic acid. https://echa.europa.eu/it/registration-dossier/-/registered-dossier/5331

[16] ARPAV (2019) Il composto cC6O4 nel Po: i monitoraggi effettuati al 23 luglio. https://www.arpa.veneto.it/arpav/pagine-generiche/il-composto-cc604-nel-po

[17] Ojo AF, Peng C, Ng JC (2020). Combined effects and toxicological interactions of perfluoroalkyl and polyfluoroalkyl substances mixtures in human liver cells (HepG2). Environ Pollut.

[18] Hoover G, Kar S, Guffey S, Leszczynski J, Sepúlveda MS (2019). In vitro and in silico modeling of perfluoroalkyl substances mixture toxicity in an amphibian fibroblast cell line. Chemosphere.

[19] Sheng N, Cui R, Wang J, Guo Y, Dai J (2018). Cytotoxicity of novel fluorinated alternatives to long-chain perfluoroalkyl substances to human liver cell line and their binding capacity to human liver fatty acid binding protein. Arch Toxicol.

[20] Coperchini F, Pignatti P, Lacerenza S (2015). Exposure to perfluorinated compounds: in vitro study on thyroid cells. Environ Sci Pollut Res Int.

[21] Pirali B, Negri S, Chytiris S (2009). Perfluorooctane sulfonate and perfluorooctanoic acid in surgical thyroid specimens of patients with thyroid diseases. Thyroid.

[22] Vermes I, Haanen C, Steffens-Nakken H, Reutelingsperger C (1995). A novel assay for apoptosis. Flow cytometric detection of phosphatidylserine expression on early apoptotic cells using fluorescein labelled Annexin V. J Immunol Methods.

[23] Kroemer G, Dallaporta B, Resche-Rigon M (1998). The mitochondrial death/life regulator in apoptosis and necrosis. Annu Rev Physiol.

[24] Faleiro L, Lazebnik Y (2000). Caspases disrupt the nuclear-cytoplasmic barrier. J Cell Biol.

[25] Crowley LC, Christensen ME, Waterhouse NJ (2016). Measuring survival of adherent cells with the colony-forming assay. Cold Spring Harb Protoc.

[26] Devasagayam TP, Tilak JC, Boloor KK, Sane KS, Ghaskadbi SS, Lele RD (2004). Free radicals and antioxidants in human health: current status and future prospects. J Assoc Physicians India.

[27] McGregor GH, Campbell AD, Fey SK (2020). Targeting the metabolic response to statin-mediated oxidative stress produces a synergistic antitumor response. Cancer Res.

[28] Berridge MV, Herst PM, Tan AS (2005). Tetrazolium dyes as tools in cell biology: new insights into their cellular reduction. Biotechnol Annu Rev.

[29] Starkov AA, Chinopoulos C, Fiskum G (2004). Mitochondrial calcium and oxidative stress as mediators of ischemic brain injury. Cell Calcium.

[30] Dröge W (2002). Free radicals in the physiological control of cell function. Physiol Rev.

[31] Adam-Vizi V, Chinopoulos C (2006). Bioenergetics and the formation of mitochondrial reactive oxygen species. Trends Pharmacol Sci.

[32] Turrens JF (2003). Mitochondrial formation of reactive oxygen species. J Physiol.

[33] Valko M, Leibfritz D, Moncol J, Cronin MT, Mazur M, Telser J (2007). Free radicals and antioxidants in normal physiological functions and human disease. Int J Biochem Cell Biol.

[34] Giorgio M, Trinei M, Migliaccio E, Pelicci PG (2007). Hydrogen peroxide: a metabolic by-product or a common mediator of ageing signals?. Nat Rev Mol Cell Biol.

[35] Afanas'ev IB (2007). Signaling functions of free radicals superoxide & nitric oxide under physiological & pathological conditions. Mol Biotechnol.

